# Formulation and nutritional evaluation of a healthy vegetable soup powder supplemented with soy flour, mushroom, and moringa leaf

**DOI:** 10.1002/fsn3.476

**Published:** 2017-05-03

**Authors:** Tasnim Farzana, Suman Mohajan, Trissa Saha, Md. Nur Hossain, Md. Zahurul Haque

**Affiliations:** ^1^ Institute of Food Science and Technology (IFST) Bangladesh Council of Scientific and Industrial Research (BCSIR) Dhaka Bangladesh

**Keywords:** fiber, minerals, moringa leaf, Oyster mushroom, protein, soy flour, Vegetables soup powder

## Abstract

The research study was conducted to develop a healthy vegetables soup powder supplemented with soy flour, mushroom, moringa leaf and compare its nutritional facts with locally available soup powders. Proximate analysis and sensory evaluation were done by standard method. In this study, moisture, ash, protein, fat, fiber, carbohydrate, and energy content were ranged from 2.83% to 5.46%, 9.39% to 16.48%, 6.92% to 16.05%, 4.22% to 6.39%, 0.22% to 1.61%, 58.81% to 75.41%, and 337.42 to 386.72 kcal/100 g, respectively. Highest content of vitamin D, minerals, protein, and fiber and lowest content of moisture, fat, and carbohydrate were found in the presently developed soy–mushroom–moringa soup powder compare to locally available soup powders. Vitamin C was also found significantly higher than locally available soup powders S1, S2, and S3. Heavy metals were not found in any of the soup powders. On the sensory and microbiological point of view, the presently developed soup powder was found highly acceptable up to 6 months. So, the developed soy–mushroom–moringa soup powder is nutritionally superior to locally available soup powders and sufficient to meet day‐to‐day nutritional requirements as a supplement.

## 1 INTRODUCTION

People are passing hectic life due to urbanization. They do not have enough time to cook foods and are becoming habituated to consume fast foods and something like that. Most of these foods are junk foods due to high sugar, fat, salt content, and low nutrient value in terms of protein, fiber, vitamin, and mineral content (Kaushik, Narang, & Parakh, 2011). Consumption of these nutrient‐deficient foods ultimately leads to malnutrition and related diseases. Moreover, cereal‐based dietary pattern may also exaggerate this condition. This problem could be overcome by supplying easy‐to‐cook nutrient‐enriched foods. One of the easy‐to‐cook foods that are available in our country is dried soup powder which is playing an important role in fulfilling present and future social consumer requirements (Krejcova, Cernohorsky, & Meixner, 2007). Dried soup powders have an advantage of protection from enzymatic and oxidative spoilage and flavor stability at room temperature over long periods of time (6–12 months). In addition, they are ready for reconstitution in a short time for working families, hotels, hospitals, restaurants, and institutional use as well as to military rations. Moreover, they exert light weight for shipping and availability at all time of the year (El Wakeel, 2007; Osman, El‐Damaty, Shaheen, & Ibrahim, 1991; Rekha, Yadav, Dharmesh, Chauhan, & Ramteke, 2010). However, most of the locally available soups are not up to the mark regarding nutritional quality. The nutritional quality could be improved by introducing protein, minerals, and vitamin sources from plant origin that are suitable for all types of people. Considering these, soybean**,** mushroom, and moringa leaf (*Moringa Oleifera)* would be good choice of sources owing to their high nutritional quality.

Soybean (*Glycine max*), a grain legume, is an excellent source of protein (43.2%), about 18% of oil, mainly polyunsaturated and monounsaturated fatty acids with small amounts of saturated fat, whereas most of the oilseeds contain 40%–50% oil (Van Ee, 2009), 31.3% carbohydrates (Kundu, Brahmchari, Bera, Kundu, & Roychoudhury, 2011), and adequate amounts of minerals and vitamins. On the basis of amino acids profile, soybean is superior to other plant proteins because it contains most of the essential amino acids except methionine (Nielsen, 1996), which is abundant in cereals, and high lysine and tryptophan content which is limiting in most cereals (Serna‐Saldivar, Vargas, Genzalez, Bedolla, & Medina, 1988; Waliszewski, Estrada, & Pardio, 2000). The predominant type of fat in soybeans is linoleic acid, accounting for approximately 50% of the total fat content which is beneficial for health. These remarkable properties of soybean make it an ideal choice of supplementary foods and based on this scenario various industrial sectors have been developed in different parts of the world (Mooriya, 2003).

Mushroom is considered to be a complete and safest food, suitable for all age groups. This nutrient dense versatile food can be taken as a substitute of meat, fish, fruits, and vegetables (Kakon, Choudhury, & Saha, 2012). It represents an excellent source of protein, vitamins (B1, B2, niacin, C, folic acid, and provitamin D ergosterol), dietary fibers, minerals (P, K, Na, Ca, and Fe) and is low in fat (Kurtzman, 2005; Moharram, Salama, & Hussien, 2008). On dry matter basis, protein content in mushrooms ranges from 20% to 40% (Chang & Buswell, 1996; Chang & Mshigeni, 2001; Kurtzman, 2005) and contain an abundance of essential amino acids like lysine and leucine which are limited in cereal grains (Chang & Buswell, 1996; Kurtzman, 2005; Sadler, 2003). It is a unique plant food in that they are very low in carbohydrates making them ideal for diabetic patents. Mushroom is also an excellent source of vitamin B12 (Koyyalamudi, jeong, Cho, & Gerald pang, 2009) which is generally not present in plant foods and ideal choice for the vegetarians. The balanced status of protein, fat, carbohydrate, minerals, vitamins, amino acids, and active ingredients makes it an ideal choice for food supplementation. Therefore, mushrooms can be a good supplement to cereals (Chang & Buswell, 1996) and are used in various sausages, vegetables, health drinks, soups, cake, and bakery products.

*Moringa oleifera* is now drawing a great attention throughout the world for its nutritional and medicinal value. Moringa leaves are particularly rich in tocopherols, ß‐carotene, protein, vitamins, minerals, and essential sulfur‐containing amino acids which are rarely found in daily diets (Foidl, Makkar, & Becker, 2001; Ogunsina, Radha, & Singh, 2010; Oliveira, Silveira, Vasconcelos, Cavada, & Moreira, 1999). According to Fahey (2005), vitamin C content of moringa leaves is seven times higher than that of oranges, vitamin A content is four times to carrots, calcium is four times, and protein content is two times to milk, and potassium is three times higher than that of bananas. The leaf is also rich in several antioxidant plant compounds (Sreelatha & Padma, 2009; Verma, Vijayakumar, Mathela, & Rao, 2009). Owing to these beneficial advantages of *Moringa oleifera*, its seeds, leaves, and bark are being used for the preparation of various foods like salads, juices, soups, and medicine (Foidl et al., 2001).

The reason of choosing soy flour, mushroom, and moringa leaf as supplementary ingredients is their nutritional contents which make them a complete nutritional source for regular diet. For instance, soy flour has higher protein and unsaturated fat than moringa and mushroom, whereas moringa leaf has higher fiber, minerals, and antioxidants than others. On the other hand mushroom also has higher protein, fiber, minerals, and low fat. Moreover, soy bean has lysine but no methionine, on the other hand moringa leaf has sulfur‐containing amino acids. So, all these sources complement each other and make the soup an ideal healthy food for all aged people.

Considering the above points, the present research work has been aimed to formulate a soy flour, mushroom, and moringa supplemented healthy vegetable soup powder and evaluating their nutritional and sensorial properties by comparing with locally available soups to get a better insight in this issue and at the same time to give a support to the country people a regular nutritious diet.

## 2 MATERIALS AND METHODS

The major study was carried out in the laboratory of Quality Control Research Section and microbiological study was conducted at Industrial Microbiology Research Section of Institute of Food Science and Technology (IFST), Bangladesh Council of Scientific and Industrial Research (BCSIR), Bangladesh.

### 2.1 Sample collection

Four brands of locally available soup powders were randomly selected and these were coded as S1, S2, S3, and S4. Soybean was collected from the Bangladesh Agricultural Research Institute. Oyster mushroom (*Pleurotus ostreatus*) was collected from the National Mushroom Development and Extension Center, Savar, Bangladesh. Other ingredients were collected from the local market. Soybean seeds and mushrooms were processed according to the procedure described by Farzana and Mohajan (2015).

### 2.2 Processing of moringa leaf powder

Moringa leaves were obtained from the residential area of Bangladesh Council of Scientific and Industrial Research (BCSIR), Bangladesh. The processing of moringa leaf was carried out by making modification in the method described by Gernah and Sengev (2011). After destalking and washing, the leaves were then boiled with 0.1% (v/v) sodium meta‐bi‐sulfite for 5 min and then spread out on racks for 10–15 min to drain out water. The leaves were then spread thinly on mesh and dry in solar dryer for about 4 hr (temperature range is 35°C–55°C on a very sunny day). The final product was found very brittle. The dried leaves were grinded into powder and then packaged in a translucent or colored polythene bag and kept in a plastic container with cover and stored at room temperature at 30°C ± 2 for chemical analysis.

### 2.3 Preparation and formulation of soy–mushroom–moringa soup powder

**Figure 1:**
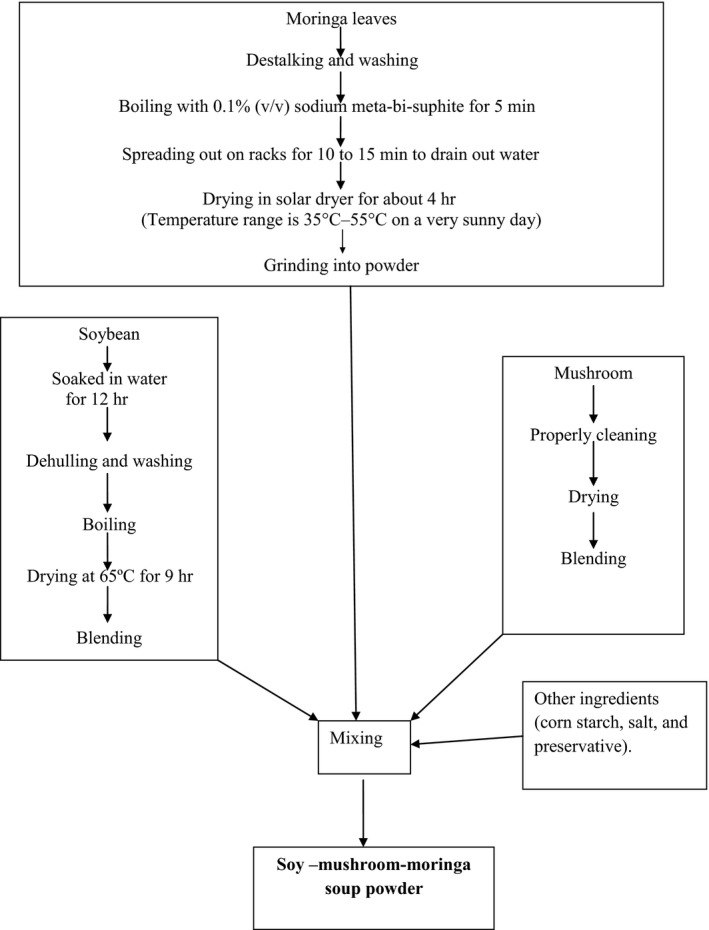
Flowchart for the preparation of soy–mushroom–moringa soup powder

Soy–mushroom–moringa soup powder was prepared by mixing of soy flour, mushroom, and moringa leaf powder with other ingredients (corn starch, salt, flavors, and preservatives). The prepared soup powders were then sealed in translucent or colored polythene bag and used for chemical analysis and sensory evaluation. For shelf life study, prepared soy–mushroom–moringa soup powder was also sealed in colored polythene bags and stored up to 9 months at room temperature. The preparation and formulation of the product has been depicted in Figure 1 and Table 1.

**Table 1:** Formulation of soy–mushroom–moringa soup powder

Ingredients	Amount (%)
Corn starch	60.5
Soy flour	10
Mushroom	5
Moringa leaf powder	8.5
Salt	16
Sodium benzoate	0.025

### 2.4 Sensory analysis

The sensory attributes including flavor, taste, texture, consistency, color, and overall acceptability were evaluated using the nine‐point hedonic‐scale scorecard by a trained 10‐member panelist selected from the staff members of the Institute of Food Science and Technology (IFST), Bangladesh Council of Scientific and Industrial Research (BCSIR), Bangladesh. Each attribute was scored based on its intensity scaled on a 9‐point hedonic scale (9 =  liked very extremely, 8 =  liked very much, 7 =  like moderately, 6 =  liked slightly, 5 =  neither liked or disliked, 4 =  disliked slightly, 3 =  disliked moderately, 2 =  disliked very much, and 1 =  disliked extremely) for color, flavor, texture, and taste.

### 2.5 Cooking procedures of soup powders

Twenty‐five gram of the newly developed soup powder was added into 350 ml water and boiled for 5–6 min, and readied for serving of two persons. In the case of locally available soup powders, for two serving sizes, 25–28 g of soup powders was added into 330–350 ml of water and boiled for 5–6 min.

### 2.6 Proximate analysis of soy–mushroom–moringa soup and locally available soup powders

The proximate composition (i.e., moisture, ash, protein, fat, fiber) of the soy–mushroom–moringa soup powder and locally available similar products were estimated according to the standard analytical methods (AOAC, 2000). The carbohydrate content was determined by calculated difference method and the energy value was determined by multiplying the proportion of protein, fat, and carbohydrate by their respective physiological energy values and taking the sum of the products (Eneche, 1999; Farzana & Mohajan, 2015).

### 2.7 Determination of vitamin D, vitamin C, and trace elements of soy–mushroom–moringa soup and locally available soup powders

Vitamin D was estimated by HPLC method (Kaushik, Sachdeva, Arora, & Wadhwa, 2014). Vitamin C was determined by Indophenol method as per the procedure as outlined by Food Analysis Laboratory Manual (Zvaigzne, Karklina, Seglina, & Krasnova, 2009). Sodium and potassium contents were determined by flame photometric method (Jahan, Gosh, Begum, & Saha, 2011; Ward & Johnston, 1962). Iron, manganese, and zinc were determined by Flame Atomic Absorption Spectrometric method (AOAC, 2005; Kirk & Sawyer, 1991)^.^

### 2.8 Determination of heavy metals of soy–mushroom–moringa soup powder

Lead, arsenic, cadmium, and mercury of soy flour, mushroom, moringa leaves, soy–mushroom–moringa soup powder were determined by Flame Atomic Absorption Spectrometric method (Kirk & Sawyer, 1991).

### 2.9 Microbial analysis of soy–mushroom–moringa soup powder

Microbial analysis especially Total Viable Count, Coliforms and *E. coli,* Yeast and Molds of soy–mushroom–moringa soup powder were carried out according to the procedure of Bacteriological Analytical Manual (Feng, Weagant, Grant, & Burkhardt, 2013; Maturin & Peeler, 2001; Tournas, Stack, Mislivec, Koch, & Bandler, 2001).

For Total Viable Count, test sample and media were prepared according to standard operating procedure. The media and test portion of the sample were mixed thoroughly and let the petridishes solidify. The inoculated petridishes were incubated invertly in the incubator at 30°C ± 1°C for 72 hr ± 3 hr. All the works were done duplicate. The result was expressed according to international methods (Maturin & Peeler, 2001).

For Coliforms and *E. coli*, test portion, initial suspension, and sufficient number of dilutions were made following the standard method. Double‐ and single‐strength Lauryl sulfate tryptose broth, EC broth, and Brilliant green lactose bile broth were made as confirmation media in McCartney bottle or screw cap tube with inverted Durham tube. Three tubes of double‐ and single‐strength liquid selective enrichment medium were then inoculated with a specified quantity of the test sample or with a specified quantity of an initial suspension and incubated at 30°C or 37°C for 24 hr or 48 hr. A series of tubes of the confirmation medium were inoculated with the cultures from the tubes of double‐ and single‐strength selective enrichment medium in which gas formation or opacity preventing the detection of gas formation has been noted. The most probable number of coliforms per milliliter or per gram of sample (i.e., the MPN) was calculated from the number of tubes in the new series showing gas formation. A table for determination of most probable numbers was used (Feng et al., 2013).

For Yeast and Molds, the Dichloran Rose Bengal Chloramphenicol (DRBC) and Dichloran 18% Glycerol (DG18) agar media were prepared according to instruction (Oxoid Ltd, Basingstroke, Hampshire, England). Serial dilutions of the sample were prepared using peptone solution. Using the procedures for the Surface Spread Method, the petridishes were prepared and inoculated. For high water activity (water activity > 0.95) foods, Dichloran Rose Bengal Chloramphenicol (DRBC) agar was used and for reduced water activity (water activity < 0.95) foods, Dichloran 18% Glycerol (DG18) agar was used. The plates were incubated upright at 25 ± 1°C for 5. DG18 plates should be incubated for 7 days. Presumptive yeast colonies were confirmed by microscopic examination of each colony type. The result was expressed according to international methods (Tournas et al., 2001).

### 2.10 Statistical analysis

Data analyses were carried out using Statistical Package for the Social Sciences (SPSS version 15.0 SPSS Inc. Chicago, Illinois, and U.S.A). Values were expressed as percentage and mean ± SD. The significance/nonsignificance of results was determined using one‐way ANOVA and Duncan test. Means were separated using *t* test.

## 3 RESULTS AND DISCUSSION

### 3.1 Chemical compositions of soy flour, mushroom powder, and moringa leaf powder (on dry basis)

The moisture, protein, fat, ash, fiber, and total carbohydrate of dehulled soy flour were found to be 1.4%, 49.3%, 24.9%, 2.8%, 3.0%, and 18.6%, respectively, in dry weight (Table 2). The fat and protein contents were found slightly higher than the results of Kundu et al. (2011) on a dry basis. In case of Oyster mushroom (*Pleurotus ostreatus*) powder, the moisture, protein, fat, ash, fiber, and total carbohydrate were found to be 4.0%, 31.8%, 2.5%, 7.0%, 12.5%, and 42.2% respectively, in dry weight (Table 2). The results were almost similar to the study of Michael, Bultosa, and Pant (2011).

**Table 2:** Proximate analysis of soy flour, mushroom, and moringa leaf powder (on dry basis)

Sample	Results					
	Moisture (%)	Ash (%)	Protein (%)	Fat (%)	Fiber (%)	Carbohydrate (%)
Soy flour	1.4	2.8	49.3	24.9	3.0	18.6
Mushroom	4.0	7.0	31.8	2.5	12.5	42.2
Moringa	4.5	9.29	31.64	6.95	11.37	60.75

In case of moringa leaf powder, the moisture (4.5%), protein (31.64%), fat (6.95%), ash (9.29%), fiber (11.37%), Iron (10.82 mg/100 g), and total carbohydrate (36.25%) were found in dry weight (Table 2) which is supported by other studies (Dachana, Rajiv, Dasappa, & Prakash, 2010; Sengev, Abu, & Gernah, 2013).

### 3.2 Chemical compositions of the developed soy–mushroom–moringa soup powder and locally available soup powders (on dry basis)

#### 3.2.1 Moisture

The moisture content of the five soup powder varied significantly. The values ranged from 2.83% to 5.46%. The lowest moisture content was found in our soy–mushroom–moringa soup powder (2.83%) and highest was found in S3 sample (5.46%) (Table 3). Moreover, the moisture content of the newly developed soup was lower than the reports of other studies (Rekha et al., 2010; Rubilar et al., 2012; Singh & Chaudhary, 2015). The lower moisture content may be due to the incorporation of soy flour and moringa leaf powder in the preparation of soup which is supported by our previous studies that increase in soy flour percentages decreases moisture content in biscuit (Farzana & Mohajan, 2015) and the study of Sengev et al. (2013) that increase in moringa leaf powder decrease moisture content of bread. This may be explained as soy flour contained a greater amount of total dry solid with high emulsifying properties compared to other flours. Furthermore, low moisture content of *Moringa* leaf powder used in the blends may also substantiate this study and might have implications in terms of the texture and microbiological quality of soup processed with added *Moringa* leaf powder (Sengev et al., 2013). Moisture content is an important factor in maintaining food quality because increase moisture facilitates the growth of microbes and ultimately destroy quality. According to Luh and Woodroof (1975), moisture content is an important factor of microorganism's growth. Microorganisms cannot grow when moisture content is below 8%. On the other hand, when moisture is above 18%, some microorganisms may be reproduced gradually. In addition, El Wakeel (2007) claims that in case of dried materials, moisture content less than 10% is considered as more proper for keeping quality of soup ingredients.

**Table 3:** Proximate analysis of soy–mushroom–moringa soup powder and four locally available soup powders (on dry basis)

Sample	Results						
	Moisture (%)	Ash (%)	Protein (%)	Fat (%)	Fiber (%)	Carbohydrate (%)	Energy (Kcal/100 g)
Soy–mushroom–moringa soup	2.83 ± 0.06^d^	16.48 ± 0.02^a^	16.05 ± 0.05^a^	4.22 ± 0.03^e^	1.61 ± 0.03^a^	58.81 ± 0.13^f^	337.42 ± 0.05^e^
S1	5.46 ± 0.08^a^	12.53 ± 0.04^c^	6.92 ± 0.07^e^	4.92 ± 0.05^c^	0.22 ± 0.01^f^	75.41 ± 0.17^a^	373.6 ± 0.05^d^
S2	3.65 ± 0.07^c^	9.39 ± 0.03^f^	13.75 ± 0.06^b^	4.35 ± 0.06^d^	0.64 ± 0.03^d^	71.86 ± 0.25^b^	386.72 ± 0.24^a^
S3	4.06 ± 0.06^b^	12.27 ± 0.04^d^	13.36 ± 0.05^c^	6.39 ± 0.08^a^	0.80 ± 0.07^c^	67.18 ± 0.30^e^	379.67 ± 0.06^c^
S4	4.05 ± 0.03^b^	11.47 ± 0.02^e^	13.43 ± 0.04^c^	5.62 ± 0.06^b^	0.39 ± 0.03^e^	69.08 ± 0.20^c^	380.62 ± 0.04^b^

Values are means of triplicates ± standard deviation. Values with the same superscript in a column are not significantly different (*p* > .05).

#### 3.2.2 Ash

The ash content of the five soups ranged from 9.39% to 16.48%. In case of soy–mushroom–moringa soup powder, it (16.48%) was highest among all other soup powders, S1 (12.53%), S2 (9.39%), S3 (12.27%), and S4 (11.47%), whereas lowest content was found for locally available S2 soup (9.39%) (Table 3). This difference was significant (*p* < .05). The ash content of the presently developed soy–mushroom–moringa soup powder was found higher than that of the results of other studies (Igwenyi & Azoro, 2014; Rekha et al., 2010; Rubilar et al., 2012). Moreover, our result is supported by the study of Singh, Ghosh, and Patil (2003). He observed higher percentage of ash (13.5%) content during the development of mushroom–whey soup powder. The highest mineral content of the newly developed soy–mushroom–moringa soup powder may be due to the supplementation of soy flour, mushroom, and moringa leaves as soy flour, mushroom, and moringa leaves are good source of minerals, supported by other studies (Ayo, Ayo, Popoola, Omosebi, & Joseph, 2014; Dachana et al., 2010; Farzana & Mohajan, 2015; Sengev et al., 2013). The higher ash content of the newly developed soup powder suggests that it is a better source of minerals.

#### 3.2.3 Protein

In this study, the protein content of the five soup powders varied significantly. It ranged from 6.92% to 16.05%. The soy–mushroom–moringa soup powder showed significantly highest protein content (16.05%) among all other locally available soups, S1 (6.92%), S2 (13.75%), S3 (13.36%), and S4 (13.43%), whereas S1 soup (6.92%) showed the least protein content. The protein content of S3 soup (13.36%) was found similar to that of S4 soup (13.43%) (Table 3). The protein content of the presently developed soy–mushroom–moringa soup powder was higher than that of the results of other studies (Rahman, Saifullah, & Islam, 2012; Rekha et al., 2010; Rubilar et al., 2012; Singh et al., 2003). The highest protein content of soy–mushroom soup powder may be was owing to soy flour, mushroom, and Moringa leaves supplementation in the soup. This result is supported by the finding of other studies where incorporation of soy flour, mushroom, or moringa leaves increases the protein content (Ayo et al., 2014; Farzana & Mohajan, 2015; Sengev et al., 2013). Soybean is a good source of protein (40%–45%) and an excellent complement to lysine‐limited cereal protein (Garg, Malik, Lule, & Awasti, 2014). Hence, this is the basis for the use of soy flour as an economical protein supplement in soup, biscuit, bread, pasta, and other cereal products (Hegstad, 2008). Mushroom is a good source of high‐quality protein (20%–40% on dry weight basis) (Singh, Kumar, & Singh, 1995). So mushrooms can be used for fortification in soups and different products. Moringa leaf is also a good source of protein (26.2%) (Dachana et al., 2010). Owing to higher protein content of these three plant sources it could be assumed that addition of soy flour, mushroom, and moringa leaf powder in soup have a greater potential in overcoming protein–calorie malnutrition of the people.

#### 3.2.4 Fat

The fat content of the five soup powders ranged from 4.22% to 6.39%. The highest fat content was found in locally available S3 soup powder (6.39%), whereas least amount was found in soy–mushroom–moringa soup powder (4.22%) (Table 3). The fat content of the newly developed soy–mushroom–moringa soup powder was not only significantly lower (4.22%) than all other locally available soups but also lower than that of the results of other studies (Igwenyi & Azoro, 2014; Rubilar et al., 2012; Singh et al., 2003). Fat content for other locally available soup powders S1, S2, and S4 was found to be 4.92%, 4.35%, and 5.62%, respectively (Table 3). The lower fat content of soy–mushroom–moringa soup may be due to minimal fat content of mushroom (1.61%–2.55%) (Michael et al., 2011) and moringa (2.4%) (Dachana et al., 2010). Soy flour contains 18% of fat (Kundu et al., 2011). The two polyunsaturated fats that are found in soy flour, including the two essential fatty acids, linoleic and linolenic, assist in the absorption of vital nutrients that are required for human health (Hegstad, 2008). The lower fat of this soup will make it an appropriate choice as a food for everybody. Moreover, a low‐fat diet can help us ward off serious medical conditions, including heart disease, high cholesterol, diabetes, etc.

#### 3.2.5 Fiber

The fiber content of the five soup powders varied significantly in this study. It ranged from 0.22% to 1.61%. The highest fiber content was found in the presently developed soy–mushroom–moringa soup (1.61%), whereas least amount in locally available S1 soup (0.22%). The fiber contents of other locally available soup powder were S1 (0.22%), S2 (0.64%), S3 (0.80%), and S4 (0.39%) (Table 3). The fiber content of the newly developed soy–mushroom–moringa soup was almost similar to the results of other studies (Abdel‐Haleem & Omran, 2014; Rekha et al., 2010; Rubilar et al., 2012). The highest fiber content in the presently developed soup perhaps because of inclusion of soy flour, mushroom, and moringa leaf powder in the preparation of soups that is supported by other studies (Dachana et al., 2010; Farzana & Mohajan, 2015; Ndife, Abdulraheem, & Zakari, 2011; Sengev et al., 2013). This makes the newly developed soup a great choice of fiber. According to well‐documented studies, dietary fiber plays an important role in the prevention of several diseases such as cardiovascular diseases, diverticulosis, constipation, irritable colon, cancer, and diabetes (Elleuch et al., 2011; Slavin, 2005). So, the presently developed soup powder may be helpful in preventing these diseases.

#### 3.2.6 Carbohydrate

In this study, the carbohydrate content of the five soups varied significantly. It ranged from 58.81% to 75.41%. The lowest carbohydrate content was found in our soy–mushroom–moringa soup powder (58.81%), whereas highest amount in locally available S1 soup powder (75.41%) (Table 3). The lower carbohydrate content of the presently developed soup powder possibly as a result of lower carbohydrate content of soy flour, mushroom, and moringa leaf powder that are used in the preparation of soup.

#### 3.2.7 Energy value

In this study, the energy value of the five soups ranged from 337.42 to 386.72 (kcal/100 g). The highest content was found for the locally available soup S2 (386.72 kcal/100 g), whereas least in the presently developed soy–mushroom–moringa soup (337.42 kcal/100 g) (Table 3). The lower value of energy in the newly developed soy–mushroom–moringa soup may be owing to lower fat and carbohydrate content.

### 3.3 Vitamin D, Vitamin C, and trace elements content

Vitamins and trace elements play an important role in maintaining proper function and good health in the human body. Inadequate intake of minerals in the diet is often associated with an increased susceptibility to infectious diseases due to the weakening of the immune system. In this study, vitamin D, vitamin C, and trace elements content of the five soup powders varied significantly. The vitamin D content of the newly developed soy–mushroom–moringa soup powder (85 μg/100 g) was significantly higher than the locally available soup powders S1 (22.5 μg/100 g), S2 (30 μg/100 g), and S3 (27.5 μg/100 g), S4 (35 μg/100 g) (Table 4). The higher vitamin D content may be due to the presence of mushroom as it is an excellent source of vitamin D (Kurtzman, 2005; Moharram et al., 2008).

**Table 4:** Vitamin D, Vitamin C and trace elements content of the soy‐mushroom‐moringa soup powder and four locally available soup powders (on dry basis)

Sample	Results						
	Vit D (μg/100 g)	Vit C (mg/100 g)	Na (mg/100 g)	K (mg/100 g)	Mn (mg/100 g)	Zn (mg/100 g)	Fe (mg/100 g)
Soy–mushroom–moringa soup	85.0 ± 0.03^a^	6.4 ± 0.03^b^	2425.1 ± 0.10^a^	288.85 ± 0.05^a^	2.06 ± 0.02^a^	3.70 ± 0.01^a^	3.82 ± 0.02^a^
S1	22.5 ± 0.01^e^	4.0 ± 0.01^d^	1296.20 ± 0.09^d^	186.95 ± 0.04^e^	0.45 ± 0.01^d^	0.95 ± 0.04^c^	1.91 ± 0.01^e^
S2	30.0 ± 0.02^c^	5.4 ± 0.02^c^	1114.32 ± 0.08^e^	261.65 ± 0.05^b^	0.44 ± 0.01^d^	0.95 ± 0.02^c^	3.02 ± 0.03^c^
S3	27.5 ± 0.02^d^	3.2 ± 0.01^e^	1417.55 ± 0.10^c^	205.64 ± 0.02^d^	0.83 ± 0.02^b^	0.84 ± 0.01^d^	2.75 ± 0.05^d^
S4	35.0 ± 0.01^b^	7.6 ± 0.04^a^	1645.22 ± 0.11^b^	229.04 ± 0.03^c^	0.71 ± 0.03^c^	1.29 ± 0.01^b^	3.47 ± 0.02^b^

Values are means of triplicates ± standard deviation. Values with the same superscript in a column are not significantly different (*p* > .05).

The vitamin C content of the presently developed soy–mushroom–moringa soup powder (6.4 mg/100 g) was significantly higher than the locally available soup powders S1 (4.0 mg/100 g), S2 (5.4 mg/100 g), and S3 (3.2 mg/100 g), but lower than S4 (7.6 mg/100 g) (Table 4). The higher vitamin C content may be due to the presence of moringa leaf as moringa is a good source of vitamin C (Fahey, 2005).

In the study, the trace elements content of the five soup powders varied significantly. The sodium, potassium, manganese, zinc, and iron content of the five soup powders ranged from 2425.1 to 1114.32 mg/100 g, 288.85 to 186.95 mg/100 g, 2.06 to 0.44 mg/100 g, 3.70 to 0.84 mg/100 g, and 3.82 to 1.91 mg/100 g, respectively (Table 4). The highest sodium, potassium, manganese, zinc, and iron content were found in the newly developed soy–mushroom–moringa soup. The potassium, zinc, and iron content are higher than the study of Obiakor–Okeke et al. (2014). Sodium content is also higher than the study of Rubilar et al. (2012). The increase in minerals content may be due to incorporation of soy flour, mushroom, and moringa leaf in the soup preparation as these are a good source of minerals (Ayo et al., 2014; Dachana et al., 2010; Sengev et al., 2013).

### 3.4 Heavy metals

Heavy metals are harmful and become toxic for health if they are taken above the limit of daily allowance recommended. In this study, heavy metals were not found in soy flour, mushroom, moringa leaf, and newly developed soy–mushroom–moringa soup powder (Table 5).

**Table 5:** Heavy metals of soy flour, mushroom, moringa leaf, and soy–mushroom–moringa soup powder

Sample	Results			
	Pb	As	Cd	Hg
Soy flour	ND	ND	ND	ND
Mushroom	ND	ND	ND	ND
Moringa leaf	ND	ND	ND	ND
Soy–mushroom–moringa soup powder	ND	ND	ND	ND

ND means: Not detected.

### 3.5 Organoleptic evaluations of soy–mushroom–moringa soup powder

In this study, sensory scores of developed soy–mushroom–moringa soup powder with regard to flavor, taste, texture, color, consistency, and overall acceptability were found to be highly acceptable as compared to that of locally available soup powder (Table 6).

**Table 6:** Sensory properties of the newly developed soup and locally available soup powders

Sample	Sensory attributes					
	Color	Texture	Flavor	Taste	Consistency	Overall acceptability
Soy–mushroom–moringa soup	8.5 ± 0.02^a^	8.3 ± 0.01^a^	8.4 ± 0.01^a^	8.6 ± 0.02^a^	8.5 ± 0.02^a^	8.5 ± 0.02^a^
S1	8.1 ± 0.01^d^	8.0 ± 0.02^d^	8.1 ± 0.01^b^	8.0 ± 0.02^e^	7.9 ± 0.03^f^	8.0 ± 0.01^d^
S2	8.0 ± 0.03^e^	8.1 ± 0.01^c^	8.0 ± 0.03^c^	7.9 ± 0.04^f^	8.0 ± 0.01^e^	8.0 ± 0.03^d^
S3	8.2 ± 0.02^c^	8.2 ± 0.03^b^	7.9 ± 0.02^d^	8.2 ± 0.04^c^	8.1 ± 0.02^d^	8.1 ± 0.02^c^
S4	8.1 ± 0.01^d^	8.0 ± 0.01^d^	7.8 ± 0.01^e^	8.1 ± 0.02^d^	8.2 ± 0.01^c^	8.1 ± 0.01^c^

Values are expressed as means ± standard deviation. Values with the same superscript in a column are not significantly different (*p* > .05).

### 3.6 Cooking procedures of the newly developed soup powder

Cooking time (5–6 min) of the newly developed soup powder is same as with the commercial one and organoleptically acceptable in quality.

### 3.7 Microbial analysis of soy–mushroom–moringa soup

In this study the microbial load of the presently developed soy–mushroom–moringa soup was assessed up to 9 months. According to Food Standards Australia New Zealand (2001), the total aerobic plate count, total yeast, and mold count were within the acceptable limit, whereas no Coliform or *E. coli* was found up to 6 months. After 6 months, the hygienic indicator organisms were gradually increased and the product quality became deteriorating (Table 7).

**Table 7:** Microbiological quality assessment of soy–mushroom–moringa soup powder

Sl No.	Test parameters	Results			
		0 days	60 days	120 day	180 days
1	Total aerobic bacteria, cfu/g	3.3 × 10^2^	2.3 × 10^3^	5.9 × 10^3^	2.7 × 10^4^
2	Total Coliforms, MPN/g	<0.3^b^	<0.3^b^	<0.3^b^	<0.3^b^
3	*E. coli,* MPN/g	<0.3^b^	<0.3^b^	<0.3^b^	<0.3^b^
4	Total yeasts and molds, cfu/g	<10^a^	<10^a^	<10^a^	9 .0 × 10^2^

a <10 indicate absence of test organisms in 1 g of sample.

b Most probable number (MPN) <0.3 indicates absence of test organisms in 1.0 g sample.

### 3.8 Comparison of cost of the soup powders

The cost of newly developed soup will vary in different countries depending on the availability of raw materials, however, as we are using locally available raw materials, the cost of the newly developed soup powder is 80% lower than the locally available soup powders.

## 4 CONCLUSION

On the basis of biochemical and sensory evaluation, the newly developed soy–mushroom–moringa soup powder is superior to all other locally available soup powders (S1, S2, S3, and S4). The least moisture content attributes that the soy–mushroom–moringa soup powder has a higher shelf life. On the microbiological point of view this soup powder is acceptable up to 6 months. It is also important to note that this soup is specifically high in protein, ash, fiber, vitamin D, vitamin C, sodium, potassium, manganese, zinc, and iron and low in fat and energy value which make the presently developed soy–mushroom–moringa soup powder as an appropriate choice for the fulfillment of nutritional demand of the country. This could play a great role in alleviating the protein energy malnutrition of our country.

## CONFLICT OF INTEREST

None declared.
